# Antitumor Effects of Dietary Compounds

**DOI:** 10.3390/nu15102349

**Published:** 2023-05-17

**Authors:** Zeeshan Ahmad Bhutta, Kyung-Chul Choi

**Affiliations:** Laboratory of Biochemistry and Immunology, College of Veterinary Medicine, Chungbuk National University, Cheongju 28644, Republic of Korea; doctorzeeshan94@gmail.com

Cancer is reported to be a major cause of death worldwide, accounting for 10 million in 2020 based on 19.3 million reported cases [1]. Currently, cancer treatment relies on chemotherapy, endotherapy, immunotherapy, radiotherapy, and surgical interventions depending on the type of cancer. Despite the treatment options mentioned above, which aid significantly in extending the lifespan of individuals, they result in unbearable side effects such as alopecia, anemia, dementia, nausea, peripheral neuropathy, etc. Furthermore, these traditional chemotherapeutic regimens are becoming nugatory against cancer cells because of the development of resistance to chemotherapeutic agents in cancers. Therefore, there is a dire need to search for alternatives to these traditional chemotherapeutic agents.

Plants have been used to treat all kinds of illnesses for centuries. The oldest written evidence of the use of plants as medicines, found on a Sumerian clay slab that contains recipes for different plants such as mandrake, henbane, and poppy as drugs, was found in the city of Nagpur, India, dating back 5000 years ago [2]. With the advancement of technology, bioactive compounds isolated from plants are now being used as therapeutic agents against a wide range of diseases, including cancer. It is estimated that more than 50% of the anti-cancer drugs currently being used in chemotherapy are derived from natural sources such as taxanes and their analogs, camptothecin and its derivates, vincristine, vinblastine, etc. [3]. Simply put, natural bioactive compounds are and will be a source of novel chemotherapeutic drugs used to treat various carcinomas.

A study published in this Special Issue highlights the role of Punicic acid (PunA), a type of conjugated linolenic acid (CLnA), obtained from various plant seed oils against the human colorectal HCT-116 and hypopharyngeal FaDu cancer cell lines. PunA was able to cause ferroptosis in carcinoma cells both alone and in combination with docosahexaenoic acid (DHA), a known anti-cancerous polyunsaturated fatty acid. This study also demonstrated a rise in lipid peroxidation in HCT-116 and FaDu carcinoma cells after PunA treatment [4]. Although chemicals present in plants are reported to be effective against many cancers, several studies have reported that a combination of different compounds is a better choice of method to stop cancer growth and metastasis. A recent study evaluated the efficacy of phenethyl isothiocyanate (PEITC), indole-3-carbinol (I3C), xanthohumol (X), and resveratrol (RES) against hepatocellular carcinoma (HCC). This study proved that combination therapy was an effective approach that resulted in ROS generation by NQO1. Moreover, COX-2 expression was suppressed in HepG2 cells treated with X and PEITC, while suppression in NF-κB was recorded through the Nrf2 signaling pathway. All of this leads to the start of apoptosis in HepG2 cells [5].

Corso et al. [6] reviewed the effects of different polysaccharides against breast cancer cell lines. Most of the polysaccharides used in this systemic review of studies from 2019 to 2020 used algae as their primary source for the experimental analysis against breast cancer, followed by mushrooms, plants, fruits, fungi, bacteria, and sea animals, respectively. Fucoidan and β-glucans have been most widely studied as representative polysaccharides by researchers, and it has been reported that these products can cause effects including cell death, apoptosis, the inhibition of angiogenesis, and immune system suppression through various pathways. However, the studies lack data concerning bioavailability and pharmacokinetics.

Like polysaccharides, glycosides were also reported to have anti-cancer effects on many cancers. Iridoids, a subtype of glycosides, recently attracted researchers’ attention due to their excellent effects against cancer development and metastasis [7]. Iridoids have been able to stop the proliferation, angiogenesis, and epithelial mesenchymal transition (EMT) of different cancer cell lines. Similarly, Punicalagin, a flavonoid obtained from pomegranate, has been reported in this Special Issue to inhibit the growth of osteosarcoma, glioma, cervical, ovarian, breast, colorectal, thyroid, and lung cancers [8].

In summary, each year, millions of people die because of cancer. Unregulated cell growth leads to improper functioning of the physiological functions of the body, which leads to various symptoms and results in death. This is why it is now essential to find new and better treatment alternatives to chemotherapy that can reduce the burden of deaths from hospitals and stabilize the financial circumstances of human populations and governments that are spending a tremendous amount of money on the prevention and treatment of cancer. Even though the abovementioned dietary compounds were observed to have antitumor activities, they had some drawbacks that require the immediate attention of researchers and must be solved before they can be applied in clinical trials to study their effectiveness in humans. These limitations include but are not limited to poor aqueous solubility, low penetration of the targeted tumor cells, and limited therapeutic potential with toxic side effects. Possible interventions could aim to cause these natural compounds to attach to nano-drug carriers [9] or to use them in combination [5], as reported in this Special Issue. Their effectiveness has also been reported to increase when combined with traditionally available chemotherapeutic agents such as paclitaxel, cisplatin, vincristine, and irinotecan [10].

The studies featured in this Special Issue, together with the widely available scientific literature, are critical for strengthening our understanding of dietary compounds as antitumor agents. With advancements in technology, our knowledge about the underlying biological mechanisms of these dietary compounds will increase, and we will be able to better understand, implement and evaluate these compounds for use against many tumors. These findings will assist not only researchers in developing better drugs but also healthcare professionals in educating their patients to use natural nutrient sources in order to remain healthy.
